# Effect of Twisted and Coiled Polymer Actuator (TCPA) on Crack Dispersion Properties of HPFRCC

**DOI:** 10.3390/ma15165701

**Published:** 2022-08-18

**Authors:** Takatsune Kikuta, Tomoya Nishiwaki

**Affiliations:** 1 Department of Architecture, Tohoku Institute of Technology, Sendai 982-8577, Japan; 2 Department of Architecture and Building Science, Tohoku University, Sendai 980-8579, Japan

**Keywords:** twisted and coiled polymer actuator (TCPA), artificial muscle, high performance fiber-reinforced cementitious composites (HPFRCC), crack dispersion, shrinkage, temperature, electrical stimulation, fishing line, nylon fiber

## Abstract

To achieve high durability and excellent mechanical performances of cementitious materials, research on fiber-reinforced cementitious composites (FRCC) containing various fibers has been actively conducted. On the other hand, in robotics and other fields, research on artificial muscles using Twisted and Coiled Polymer Actuator (TCPA), which have similar functions to human muscle fibers, has attracted much attention. In this study, use of this TCPA as a reinforcing fiber in high performance FRCC (HPFRCC) was proposed. The employed TCPA has a structure of coiled nylon fibers with wrapping stainless-steel fibers. The effect of the TCPA and its shrinkage motion on the crack dispersion properties of HPFRCC was investigated. The experimental results showed that the strain-hardening with multiple cracks in HPFRCC continued up to more than 7% of the ultimate strain when the TCPA was electrically stimulated to shrinkage motion. This information indicates that the TCPA has high potential to further improve HPFRCC performance.

## 1. Introduction

Concrete is the most widely used construction material in the world. Concrete has many excellent properties, e.g., high compressive strength, durability, economic efficiency, and easy availability of local raw materials. However, several drawbacks also exist. Concrete shows low tensile strength and low strain capacity, making it a brittle material. Due to these poor mechanical properties, cracks cause durability and safety problems from a long-term perspective. In recent years, there have been approaches to inhibit crack propagation at the nanoscale using nanomaterials [1,2]. This improves both the tensile strength, shrinkage, and durability of concrete. Fiber-reinforced cementitious composites (FRCC) are one of the solutions to compensate for this weakness and transform concrete into tough materials with high strain capacity [3]. A lot of studies regarding FRCC have already been conducted [4]. High-Performance Fiber Reinforced Cementitious Composites (HPFRCC) are characterized by strain-hardening behavior and multiple cracking after the first crack initiation [5]. HPFRCC is expected to provide long-term durability due to its resistance to localized cracking [6] and is being applied to the construction market [7].

The strain-hardening property of HPFRCC is due to a mechanism in which the bridging force of the dispersed fibers in the mortar matrix causes crack refinement and multiple cracks [4]. For example, fracture mechanics and micromechanics have been introduced in the development of Engineered Cementitious Composites (ECCs), which are pseudo-strain hardening cementitious materials. This allows composites to be designed based on parameters such as fiber length, fiber diameter, fiber strength, matrix fracture toughness, and fiber-matrix interfacial adhesion strength [8]. Therefore, the characteristics and performance of the fibers are essential factors. Focusing on these reinforcing fiber materials, research and development of new fiber materials with diverse performance and functions is currently undergoing in various fields. In particular, research on artificial muscle fibers, which have functions similar to muscle fibers in the human body, has received attention [9,10].

Artificial muscle fibers are actuators that expand and contract under some external control and perform work. The major classifications based on the driving source include electrochemically driven polymers [11,12,13,14,15,16,17,18,19,20,21], thermally driven shape memory polymers [22,23,24,25,26], fluid-driven pneumatic rubber [27,28,29], etc. Several problems have been identified with some of these artificial muscle fibers, including controllability, premature degradation due to repetitive motion, and material cost. However, Haines et al. [9,10] developed a novel artificial muscle fiber, called twisted and coiled polymer actuator (TCPA), which showed high output and long life. TCPA can be easily made by introducing torsion to low-cost, high-strength polymer fibers such as fishing lines and sewing thread. This new method has attracted much attention.

In cementitious materials, several studies using artificial muscles-like materials have been conducted [30]. Shape memory polymers (SMPs) are materials that have the function and characteristic of recovering their original shape when heated above glass temperature or melting temperature, even if deformed by an external force after the molding process. It is known to have better functions than general resin materials, is less expensive, and has a higher rate of shape change than shape memory alloys and other materials. Thermally driven SMPs [31,32,33,34] have been attempted to control cracking to improve long-term durability [35,36,37,38]. These studies showed that crack width could be controlled using SMPs [39]. However, there are some problems, such as the suitable heating method and the long time required for SMP actuating. In addition, the relationship between HPFRCC, which has good long-term durability, has rarely been studied between its characteristic crack dispersion and the compressive stress delivered from SMP ties.

Based on this background, this study combines HPFRCC and TCPA as a new approach in controlling the cracking of cementitious materials. This study aims to control the crack width and mechanical performance of HPFRCC by applying external electrical heat to TCPA. The method involves embedding the externally controlled TCPAs in the mortar matrix. By shrinking the embedded TCPA, the compressive stress at the cracked surface can be controlled. The experimental investigation is conducted to determine the effect of the crack width control and ductility of HPFRCC.

## 2. Experimental Overview

In this study, three types of experiment were conducted. The first is the evaluation of the TCPA’s performance; the relationship between the amount of shrinkage and temperature was evaluated because the shrinkage behavior of TCPA changes with temperature. The second is the uniaxial tensile test of HPFRCC with embedded TCPA to evaluate the relationship between shrinkage motion of TCPA and crack dispersion. The third is experiments to evaluate the effect of TCPA on the fiber bridging law at a single cracked surface, which is one of the most important material properties of HPFRCC.

### 2.1. Materials

Materials used for this study are early-strength Portland cement, silica fume, high-range water-reducing admixture, cellulosic thickener, and fine silica sand. Polyvinyl alcohol (PVA) fiber was used together. Table 1 shows the detailed information of the employed materials. In this study, TCPA was made using nylon fishing line with a diameter of 870 μm, referring to the study by Heines et al. [12]. The TCPA is currently being studied in a variety of materials. Electrothermally driven shape-memory metal wires can contract fast and deliver large strokes under heavy loads, but are expensive and hysteretic, which makes them difficult to control [40]. Thermally powered shape-memory polymers have low work capacity unless they are fiber-reinforced [41,42], and giant-work-capacity polymer/carbon nanotube (CNT) composite fibers must be redrawn between cycles [43]. On the other hand, Heines et al. reported that low-cost TCPA can be fabricated by processing nylon fibers into coils [9]. Extremely twisted nylon fibers can generate more than 100 times the force of human muscle fibers. This TCPA works by forcibly rearranging the molecules inside the fiber by continuously twisting it, and then by applying heat energy, the force of the molecules trying to return to their original arrangement is replaced by the contraction movement of a spring, which functions as a TCPA. In this study, TCPA made by twisting this nylon was employed.

### 2.2. Mix Proportion

Table 2 shows the mix proportion used in the experiment. The volume content of 8 mm PVA fiber was set at three levels: 1.5 Vol.%, 1.75 Vol.%, and 2.0 Vol.%. The water binder ratio was set in 0.45 and the sand-binder ratio was set to 0.4. Moreover, the viscosity agent was mixed in 0.003 to prevent the fiber from balling up.

### 2.3. TCPA Producing Method

**Figure 1:**
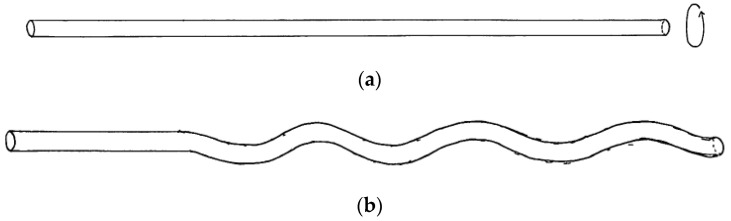

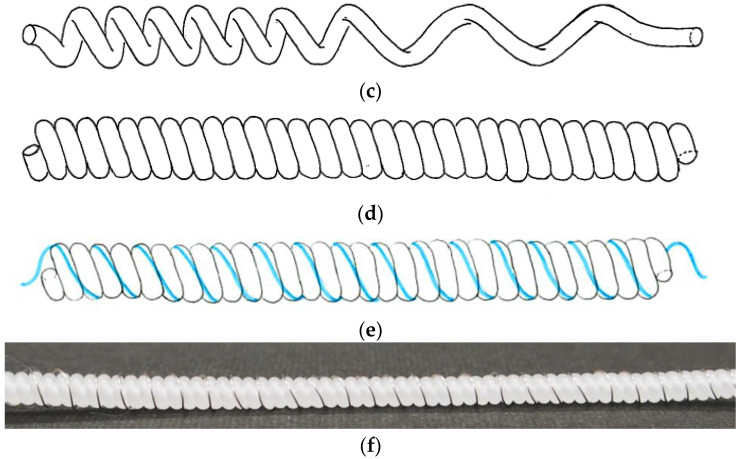
Order of creation of TCPA with nylon fishing line. (**a**) Nylon fishing line cut to specified length. (**b**) Twisting with tension acting on the fiber. (**c**) Coiled from fiber ends. (**d**) Continued torsion results in a coiled shape over the entire fiber length. (**e**) Wrap stainless steel fiber around TCPA for heating. (**f**) Completed TCPA.

As shown in Figure 1, a tensile load was applied to the nylon fiber and twisted until the entire fiber was coiled. When the nylon fibers were coiled, the surface of the fibers was heated with a heat gun to fix the spiral structure of the fibers. In addition, a stainless-steel wire with a diameter of 190 μm was wrapped around the TCPA for the purpose of actuating the TCPA with thermal energy.

### 2.4. TCPA Performance Evaluation Test 

**Figure 2:**
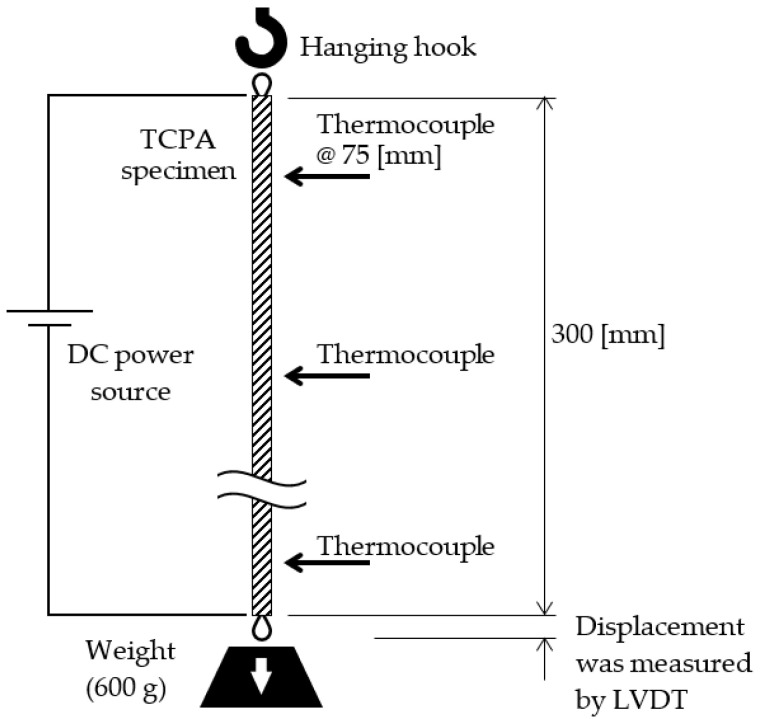
Evaluation method of heating temperature and shrinkage of TCPA alone.

Specimens shown in Figure 2 were produced to examine the influence of temperature on shrinkage of the TCPA. A DC current was applied to a stainless-steel wire and the temperature was measured by several thermocouples to measure the surface temperature and shrinkage of TCPA.

### 2.5. Uniaxial Tension Test

**Figure 3:**
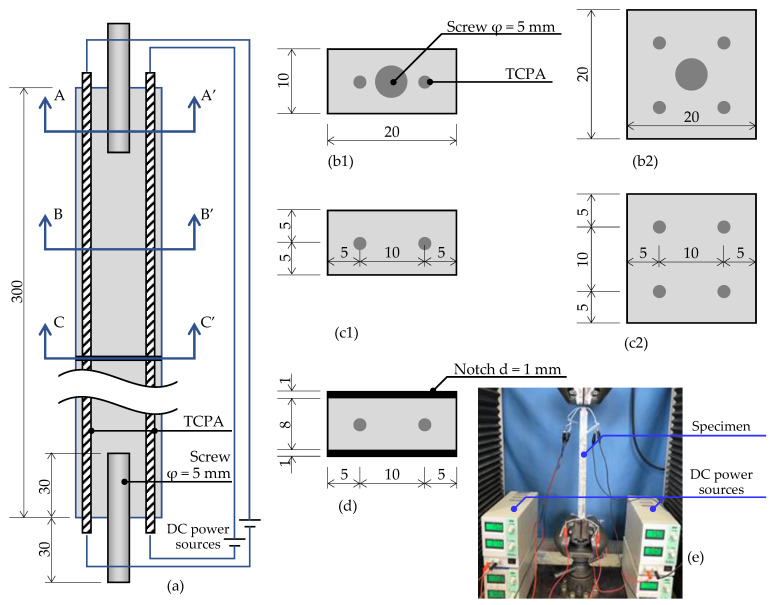
Set-up of uniaxial tension test with embedded TCPA. (**a**) Drawing of Specimen. (**b1**) A-A’ section for Test A and C. (**b2**) A-A’ section for Test B. (**c1**) B-B’ section for Test A and C. (**c2**) B-B’ section for Test B. (**d**) C-C’ section for Test C. (**e**) Test setups for Test B.

Uniaxial tensile test was performed to evaluate the stress–strain relationship of HPFRCC with embedded TCPA. Specimens with three different cross-sectional shapes were employed. The details of the test specimens are shown in Figure 3a. In Test A, the uniaxial tensile test on the specimen with the dimensions of 20 mm in width and 10 mm in thickness and two embedded TCPAs were carried out (see Figure 3b1,c1). Screws with 5 mm diameter were embedded in both ends of the specimen for the purpose of attaching the specimen to the testing machine. The uniaxial tensile test was conducted while applying a current of 15 V to TCPA. The test was executed by the displacement control with a universal testing machine of 10 KN in the maximum capacity.

In Test B, specimens with different cross-sectional geometries were prepared to evaluate the change in the stress–strain relationship and the multiple crack behavior as a function of the temperature (voltage) applied to the TCPA. The cross-section of the specimens were 20 mm square and four TCPAs embedded (see Figure 3b2,c2). The four TCPAs were each connected to independent DC power supplies, and the four TCPAs were controlled so that the same voltage was applied to all four TCPAs (see Figure 3e). The screw installation at both ends of the specimen and the test apparatus are the same as Test A.

In Test C, to evaluate the effect of TCPA on the fiber bridging law at a single crack face, a notch was introduced into the center of the specimen as shown in Figure 3d. The notches were sawed using a diamond concrete with 1 mm depth. The other geometries of the specimen were the same as Test A. Here, the mix proportions with 2.0 vol.% of PVA fiber was employed as shown in Table 2. During loading, the crack mouth opening displacement (CMOD) was measured with LVDTs placed on either side of the specimen. The applied heating on the TCPA was employed as the experimental parameter. Twenty specimens were tested in each condition. The results were used to evaluate the effect of TCPA on the fiber bridging behavior of a single cracked surface.

### 2.6. Definition of Ultimate Strain

**Figure 4:**
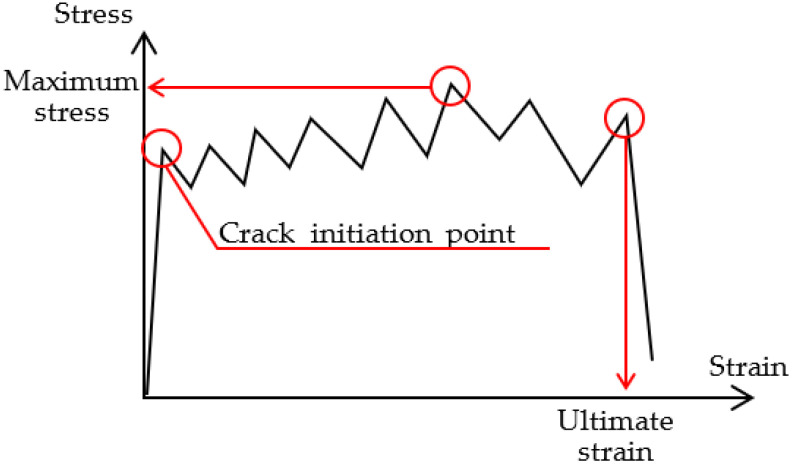
Definition of maximum strength and ultimate strain.

Figure 4 shows the definition of the mechanical characteristic value in the stress–strain relationship. The ultimate strain was defined as the point that shifted from the strain hardening behavior to the softening behavior.

## 3. Experimental Results and Discussion

### 3.1. Temperature and Shrinkage Strain Relationship of TCPA

**Figure 5:**
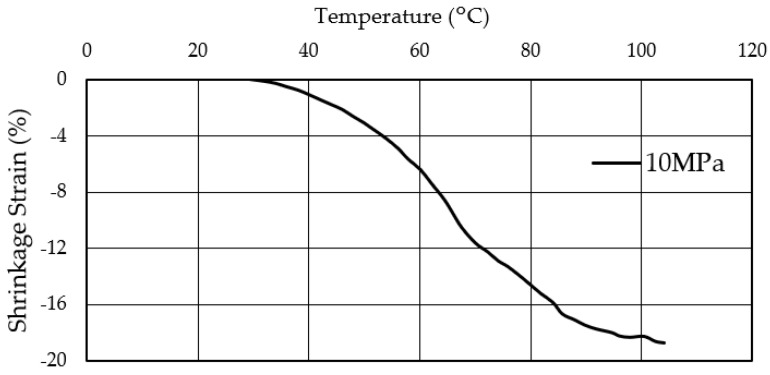
Surface temperatur−shrinkage strain relationship of TCPA.

TCPA is a material that shrinks when heated and returns to its original state when cooled. To obtain the basic performance of the TCPA used in this study, shrinkage behavior of the employed single TCPA was measured during its temperature change. The relationship between the surface temperature and the shrinkage strain of the single TCPA is shown in Figure 5. This test was conducted with tensile stress of 10 MPa applied to the TCPA. In other words, the test is started from a state in which tensile strain is applied in TCPA beforehand. This strain is referred to as the initial strain state. From the results shown in Figure 5, the shrinkage strain was gradually increased up to 3–4% from the room temperature (22 °C) to 45 °C of the surface temperature of the TCPA. After that, the shrinkage strain increased rapidly when the surface temperature exceeded 45 °C. At a surface temperature of around 100 °C, a shrinkage strain of about 18% was measured. It is clear from this result that there is a clear correlation between the shrinkage strain of TCPA and the surface temperature. It is clear that the larger the surface temperature of TCPA, the greater the shrinkage strain, as long as the temperature range is up to about 100 °C, which is acceptable for concrete materials.

### 3.2. Influence of TCPA on the Toughness of HPFRCC, Test A

**Figure 6:**
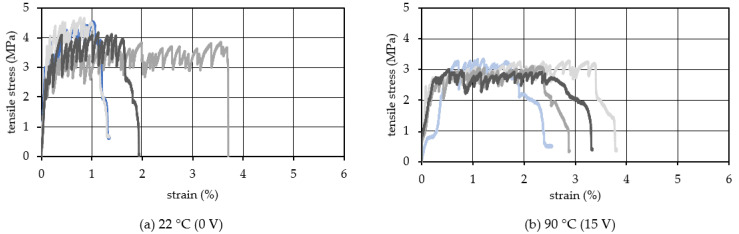
Tensile stress−strain relationship (Vf: 1.50 vol.%).

**Figure 7:**
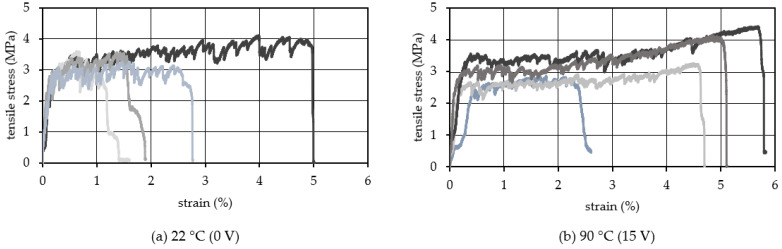
Tensile stress−strain relationship (Vf: 1.75 vol.%).

**Figure 8:**
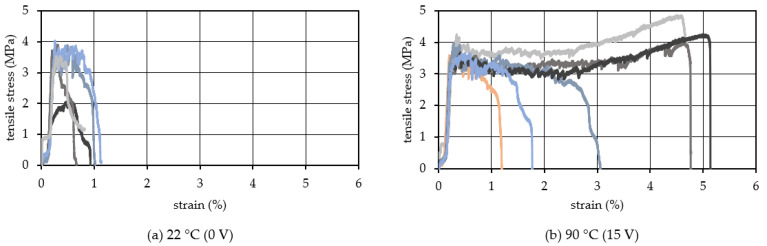
Tensile stress−strain relationship (Vf: 2.00 vol.%).

The uniaxial tensile test was conducted on the specimens with TCPA embedded in HPFRCC to clarify the effect of TCPA on the ductility performance of HPFRCC. The experiments were conducted at two levels of surface temperature: 22 °C (room temperature) and 90 °C, to control the shrinkage strain of TCPA. A voltage of 15 V was applied to the stainless-steel fibers placed on the TCPA surface to heat. The amount of PVA fiber mixed into the HPFRCC was set at three levels: 1.50, 1.75, and 2.00 vol.%. Six specimens were used for the test under each condition, as shown with different colors in Figure 6, Figure 7 and Figure 8. It should be noted that although six specimens were tested, some specimens slipped at the screw during the tensile test. Therefore, the number of curves is different in Figure 6, Figure 7 and Figure 8 because some inappropriate data was removed. Figure 6 shows the relationship between strain and stress of HPFRCC with 1.50 vol.% of PVA fibers. Figure 6a shows the results when the surface temperature of TCPA was 22 °C (room temperature, the applied voltage was 0 V). The ultimate tensile strain offers a range of 1.0 to 3.5%. The average tensile ultimate strain was 2.0%. Moreover, Figure 6b shows the test results with the TCPA with the shrinkage strain at the surface temperature of 90 °C (applied voltage was 15 V). It was confirmed that the strain hardening behavior continued to a larger deformation range compared to the specimen without heating of the TCPA (Figure 6a). On the other hand, comparing the tensile stress, the maximum tensile stress decreased by approximately 1.1 MPa when the surface temperature of TCPA was heated to 90 °C. This phenomenon can be explained by two factors. According to Mechtcherine et al. [44], the initial cracking stress and tensile strength of PVA-SHCC decrease at 60 °C. On the other hand, the toughness has been increased by the thermally reduced stiffness of the fibers. This is thought to be due to the heated TCPA making temperature high throughout the specimen, and the high temperature causes the bridging PVA fibers against cracks softening. Furthermore, as pointed out by Rokugo et al. [45], when a non-uniform temperature distribution is generated inside the specimen, tensile force is generated on the surface of the specimen with relatively lower temperature. It is inferred that heating the TCPA with a voltage of 15 V generated such a temperature distribution in the specimen. As a result, the crack initiation strength of the heated specimen shown in Figure 6b is lower than the test result at room temperature shown in Figure 6a. This trend is also observed in Figure 7b. In addition, the continuation of strain hardening behavior was not sufficient compared to Figure 7b and Figure 8b due to the small Vf: 1.5 vol.% and insufficient crack bridging. Therefore, the maximum tensile stress is considered to have decreased.

Figure 7 shows the results with the 1.75 vol.% of PVA fiber. When the TCPA was not heated, the average ultimate tensile strain was 2.5%. In contrast, when the TCPA was heated to 90 °C (the applied voltage of 15 V) and shrunk, the average tensile ultimate strain was 4.5%. It was confirmed that the strain hardening behavior continued to a significantly larger deformation range compared to the test specimens without heating. There is no significant change in the tensile stress values appeared in Figure 6, Figure 7 and Figure 8. It can be attributed to the compressive stress generated by the TCPA shrinkage is very small (0.1~0.2 MPa) relative to the tensile stress generated in the mortar cross section during the tensile test. We also note the repetitive cracking and the associated load reduction behavior in the strain hardening region after the initial cracking. In the case of no heating, the load drop associated with the occurrence of a single crack is relatively large, ranging from 0.3 MPa to 0.6 MPa. In contrast, when the specimen is heated to 90 °C and subjected to shrinkage as shown in Figure 7b, the load drop associated with one crack is 0.2 MPa to 0.4 MPa, and the load drop associated with the crack is relatively small. In other words, it can be confirmed that a more stable strain hardening behavior continues. This may be due to the shrinkage behavior of TCPA suppressed the expansion of the width of the micro-crack during generating the multiple cracks, and the stress burden of TCPA brought extra force to the PVA fibers bridging the crack surface. As a result, the crack localization was suppressed and the strain hardening behavior was more continuous. According to Ali et al. [46], the shape memory alloy in ECC can enhance the impact energy. This suggests that a synergistic effect exists between the PVA fibers which bridge the cracks, and the TCPA which drives the crack closure stress.

Figure 8 shows the results with the 2.00 vol.% of PVA fiber. The average ultimate tensile strain of TCPA shown in Figure 8a without heating was remarkably small, at 0.6%. This is due to the excessive amount of PVA fibers and the lack of sufficient fiber dispersion in the HPFRCC. Porosity increased and bond strength between the fibers and the mortar matrix reduced [47]. On the other hand, Figure 8b shows the results with the shrunk TCPA by heating to 90 °C. The average ultimate tensile strain was 3.3%, and the strain hardening behavior continued to a significantly larger deformation range compared to the case without heating. This is the same trend as the result of 1.75 vol.% PVA fiber content as shown in Figure 7. However, sufficient data regarding bond strength were not obtained within this experimental campaign. This is one of the future research issues.

These results indicate that the shrinkage strain behavior of TCPA has a positive effect on the tensile properties of HPFRCC at the fiber incorporation ratio where multiple cracks and strain hardening behavior, which are the characteristics of HPFRCC, are observed.

### 3.3. Influence of Heating Temperature of TCPA on Crack Distribution of HPFRCC, Test B

**Figure 9:**
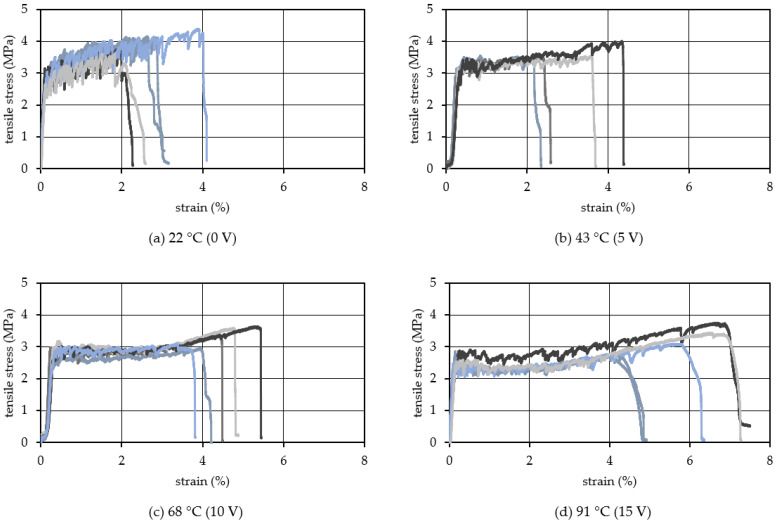
Tensile stress strain relationship (Vf: 2.00 vol.%).

**Figure 10:**
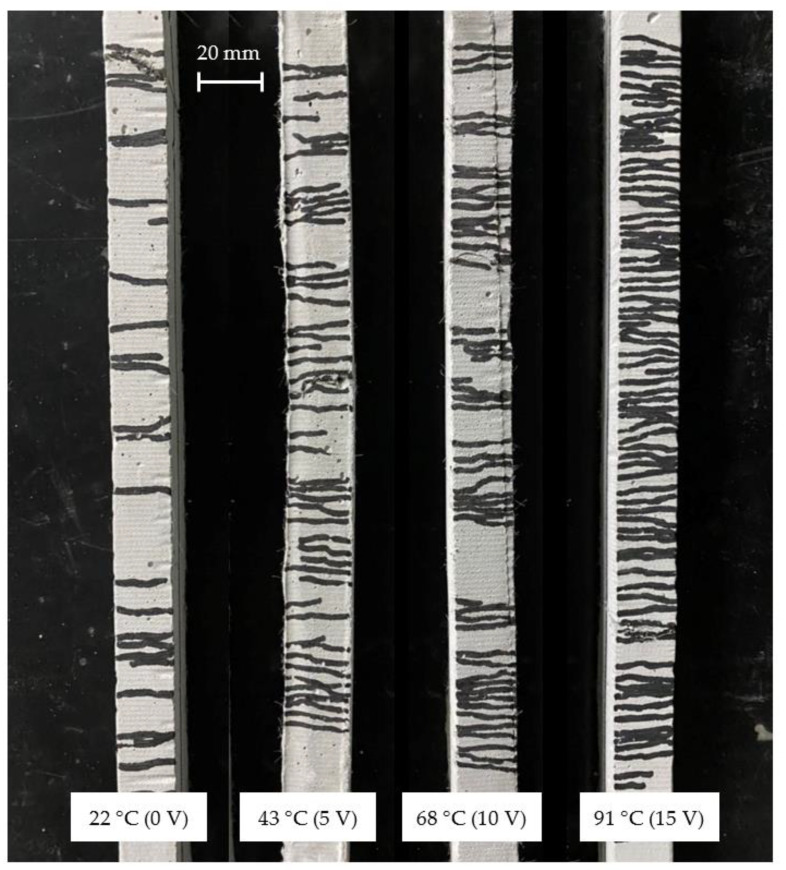
Surface temperatur−shrinkage strain relationship of TCPA.

**Figure 11:**
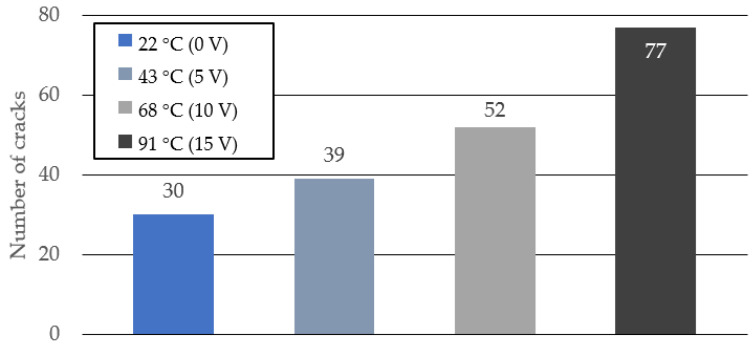
Surface temperatur−shrinkage strain relationship of TCPA.

Figure 9 shows the tensile stress–strain relationship when the voltage applied to the TCPA is varied in four steps of 0, 5, 10, and 15 V. The corresponding surface temperature of the TCPA is 22 °C (room temperature), 43 °C, 68 °C, and 91 °C, respectively. The different color lines in this figure show the results from different specimens in the same manner in Figure 6, Figure 7 and Figure 8. The specimen with four TCPAs embedded in a cross-sectional size of 20 × 20 mm shown in Figure 3c2 was used for the test here. The amount of PVA fiber incorporation is kept constant at 2.00%. From the results shown in Figure 9, it is observed that the tensile stress tends to decrease with increase of the applied voltage on the TCPA and the surface temperature. As described in the previous section, this may be due to the softening of the PVA fibers that cross-link the cracks by heating the entire test specimen as the surface temperature of the TCPA increases. Comparing the tensile endpoint strain, when the surface temperature of the TCPA was at room temperature, the average tensile endpoint strain was about 0.6% and almost no strain hardening behavior was observed. On the other hand, as the voltage was applied to the TCPA and the surface temperature was increased, the average tensile endpoint strain tended to increase: 2.1% at 43 °C (Figure 9b, applied voltage: 5 V), 3.3% at 68 °C (Figure 9c, applied voltage: 10 V), and 4.0% at 90 °C (Figure 9d, applied voltage: 15 V). 15 V). Figure 10 shows a photograph of the cracks in the specimen after the tensile test. It is clear from this photograph that the number of cracks in the specimen increases when the surface temperature of TCPA is increased to provide large shrinkage strain. Figure 11 shows the average number of cracks measured under each condition. It can be seen that there is a strong correlation between the surface temperature of TCPA and the number of microcracks generated. This can be attributed to the fact that there is a correlation between the surface temperature and the amount of shrinkage strain in the TCPA, and, thus, increasing the surface temperature increases the force in the compression direction that suppresses the expansion of the width of microcracks in the HPFRCC.

Compared to the tensile force in the cross section, the compressive prestress induced by TCPA is negligible. On the other hand, the compressive force due to the shrinkage strain behavior of TCPA can suppress the localization with crack widening. This is thought to have caused stable multiple cracks one after another, and the strain hardening region continued for a long time.

### 3.4. Effect of Shrinkage Behavior of TCPA on One Cracked Surface, Tast C

From the experimental results up on the previous section, it was confirmed that TCPA shrink behavior by heating significantly increases the ultimate strain of HPFRCC. The number of microcracks was also shown to increase. In order to clarify the factors that led to this change in mechanical behavior, experiments were conducted to evaluate the effect of TCPA on the fiber bridging law at a single cracked surface, which is one of the most important material properties of HPFRCC.

**Figure 12:**
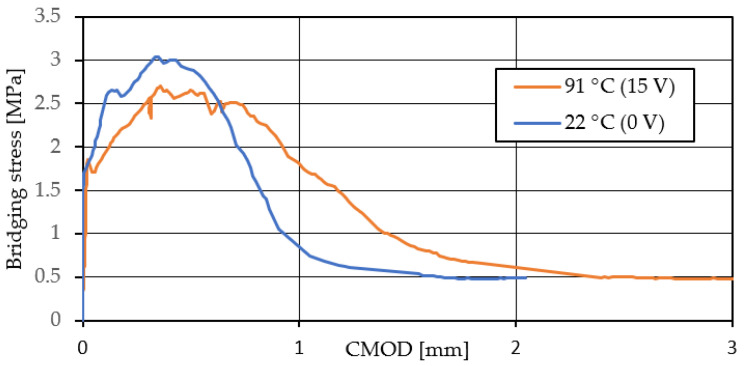
Effect of shrinkage behavior of TCPA on single cracked surface.

Figure 12 shows the relationship between the average bridging stress and CMOD obtained from the uniaxial tensile tests. The volume fraction of the PVA fiber is 2.0 vol.%. Here, CMOD is the displacement at the introduced notch. The bule curve (0 V) shows that in case of the TCPA without heating and no shrinkage, the maximum stress was 3.01 MPa with 0.38 mm of CMOD. Thereafter, the bridging stress drops sharply while the CMOD increases from 0.45 mm to 1.0 mm. On the other hand, from the red curve (15 V) with the shrinkage behavior of the TCPA, the maximum stress decreases compared to the case without heating. The reason is due to the softening of the PVA fibers that bridge the cracked surface under the influence of the heat generated by the TCPA. However, focusing on the post-peak behavior after the maximum stress, a gradual decrease in the bridging stress continues compared to the case without heating. This gradual decrease in the bridging stress continues until approximately 1.5 mm of the CMOD. In addition, focusing on the behavior near the maximum stress, when 15 V is applied to TCPA and shrinkage behavior occurs, the bridging stress remains almost unchanged from approximately 0.38 mm to 0.75 mm of CMOD after the maximum stress. 

These results suggest that the shrinkage behavior of TCPA on heating allows for stable stress-bearing up to large crack opening displacements. This is due to the additional bridging stress is available at the crack surface. As indicated in the previous section, this additional bridging force helps to ensure that multiple cracks continuously generated. In other words, the localization of cracks is reduced by heating the TCPA to generate shrinkage stress. It is clear that the shrinkage behavior of TCPA has an effect on the multiple cracking behavior of HPFRCC and on the strain hardening behavior.

## 4. Conclusions

Here, we proposed the application of TCPA, also known as artificial muscle, to HPFRCC. The findings obtained within the scope of this experiment are as follows.

1. TCPA was fabricated from nylon fiber and its performance was evaluated. A clear correlation was observed between the shrinkage strain of TCPA and the surface temperature. Within the range of about 100 °C or lower, which is acceptable for cementitious materials, a larger surface temperature of TCPA resulted in a larger shrinkage strain;
2. Specimens with TCPA applied to HPFRCC showing multiple cracks and strain hardening behavior were prepared and uniaxial tensile tests were conducted. As a result, it was clarified that the shrinkage strain generated in TCPA was effective in improving the toughness of HPFRCC;
3. By increasing the surface temperature of the embedded TCPA and increasing the shrinkage strain, the toughness of the HPFRCC was improved;
4. Prestressing in the compressive direction due to the shrinkage strain of TCPA can suppress the crack localization and crack width expansion. This was accompanied by a significant increase in the number of cracks.

## Figures and Tables

**Table 1:** Materials.

Material	Symbol	Properties
Cement	C	High early strength Portland cement, Density: 3.14 g/cm^3^
Sand	S	Density: 2.61 g/cm^3^
Fly Ash	FA	Density: 2.33 g/cm^3^
Superplasiticizer	SP	Density: 1.05 g/cm^3^
Viscosity agent	V	Polycarboxylic acid ether system, Density: 1.25 g/cm^3^
Water	W	Tap water
Polyvinyl alcohol fiber	PVA	Length: 8 mm, Diameter: 40 μm, Tensile strength: 1600 MPa, Young’s modulus: 40 GPa, Density: 1.30 g/cm^3^

**Table 2:** Mix proportions of matrix.

W/B ^1^	S/B ^1^	FA/B ^1^	SP/B ^1^	V/W	PVA vol.%
					1.50
0.45	0.4	0.3	0.009	0.003	1.75
					2.00

^1^ B: Binder (C + FA).

## Data Availability

Not applicable.
